# In Vitro and In Vivo Trypanocidal Efficacy of Synthesized Nitrofurantoin Analogs

**DOI:** 10.3390/molecules26113372

**Published:** 2021-06-02

**Authors:** Linous Munsimbwe, Anna Seetsi, Boniface Namangala, David D. N’Da, Noboru Inoue, Keisuke Suganuma

**Affiliations:** 1 Ministry of Fisheries and Livestock, Department of Veterinary Services, Mulungushi House, P.O. Box 50600, Ridgeway, Lusaka 15100, Zambia; munsimbwe@yahoo.com; 2 Unit for Environmental Science and Management, Faculty of Natural and Agricultural Sciences, North-West University, Potchefstroom 2531, South Africa; annaseetsi@gmail.com; 3 Department of Paraclinical Studies, School of Veterinary Medicine, University of Zambia, P.O. Box 32379, Lusaka 10101, Zambia; b.namangala@unza.zm; 4 Centre of Excellence for Pharmaceutical Sciences (PHARMACEN), North-West University, Potchefstroom 2520, South Africa; david.nda@nwu.ac.za; 5 OIE Reference Laboratory for Surra, National Research Centre for Protozoan Diseases, Obihiro University of Agriculture and Veterinary Medicine, Obihiro, Hokkaido 080-8555, Japan; ircpmi@obihiro.ac.jp; 6 Research Center for Global Agromedicine, Obihiro University of Agriculture and Veterinary Medicine, Obihiro, Hokkaido 080-8555, Japan

**Keywords:** animal trypanosomosis, human African trypanosomiasis, nitrofurantoin analog, trypanocidal drug

## Abstract

African trypanosomes cause diseases in humans and livestock. Human African trypanosomiasis is caused by *Trypanosoma brucei rhodesiense* and *T. b. gambiense*. Animal trypanosomoses have major effects on livestock production and the economy in developing countries, with disease management depending mainly on chemotherapy. Moreover, only few drugs are available and these have adverse effects on patients, are costly, show poor accessibility, and parasites develop drug resistance to them. Therefore, novel trypanocidal drugs are urgently needed. Here, the effects of synthesized nitrofurantoin analogs were evaluated against six species/strains of animal and human trypanosomes, and the treatment efficacy of the selected compounds was assessed in vivo. Analogs **11** and **12**, containing 11- and 12-carbon aliphatic chains, respectively, showed the highest trypanocidal activity (IC_50_ < 0.34 µM) and the lowest cytotoxicity (IC_50_ > 246.02 µM) in vitro. Structure-activity relationship analysis suggested that the trypanocidal activity and cytotoxicity were related to the number of carbons in the aliphatic chain and electronegativity. In vivo experiments, involving oral treatment with nitrofurantoin, showed partial efficacy, whereas the selected analogs showed no treatment efficacy. These results indicate that nitrofurantoin analogs with high hydrophilicity are required for in vivo assessment to determine if they are promising leads for developing trypanocidal drugs.

## 1. Introduction

African trypanosomes, transmitted by tsetse fly, cause several diseases in both humans and livestock on the Sub-Saharan African continent. Human African trypanosomiasis (HAT) is caused by *Trypanosoma brucei gambiense* and *T. b. rhodesiense.* The disease has devastating socio-economic impacts, such as reduced income generation, negative effects on the education of children, increased healthcare costs, and long-term health consequences [1,2]. In contrast, animal African trypanosomosis, which is caused by *T. congolense, T. vivax,* and *T. brucei brucei*, represents a major health concern in animal development, and its deleterious impacts can be manifested in terms of reduced livestock productivity, costly disease control, and trade implications, as well as negative impacts on agriculture and food security [3,4]. Surra is an animal trypanosomosis caused by *T. evansi* infection. It is mechanically transmitted by hematophagous biting flies such as *Tabanus* spp., *Stomoxys* spp., and species of tsetse flies [5]. Animal trypanosomoses (AT), including animal African trypanosomosis and surra, are economically important diseases in animals. They cause high mortality, low milk and meat production, poor carcass quality, reduced reproductive performance, and decreased draught animal power and manure production, as well as immunosuppression in livestock [3,5].

HAT/AT control requires an integrated approach involving the use of trypanocidal drugs [3,6], reduction in the number of vectors, and low contact between hosts and vectors [7]. Owing to the challenges in vector control programs and vaccine development, HAT/AT control primarily depends on chemotherapy using anti-parasitic agents. Currently, the nifurtimox–eflornithine combination therapy (NECT) and fexinidazole are approved for treating HAT caused by *T. b. gambiense* [8]. Particularly, fexinidazole was the first oral drug approved for HAT treatment, which shows satisfactory treatment efficacy against second-stage HAT, as compared to the nifurtimox–eflornithine combination therapy [9]. In contrast, no orally administered and safe drugs are available against HAT caused by *T. b. rhodesiense* and AT. Therefore, the screening and development of safe and effective compounds for treating HAT/AT are urgently required.

Nitrofurantoin is a hypoxic agent with activity against a myriad of anaerobic pathogens. This nitrofuran compound contains a Schiff base derived from 5-nitrofuraldehyde, which is known to be effective against a wide spectrum of gram-positive and gram-negative bacteria and various pathogens, including trypanosomes [10,11]. Nitrofurantoin analogs show good safety profiles, enhanced anti-mycobacterial potency, improved lipophilicity, and a reduced protein-binding affinity. Nitrofurans are broad-spectrum redox-active antibiotics, with dose-dependent bacteriostatic or bactericidal activity [10,12], and have been used in animal feeds, pharmaceuticals, and other applications [11,13].

The clinical drug furazolidone belongs to the group of nitrofuran antibiotics and has been widely used as an antibacterial and antiprotozoal feed additive for poultry, cattle, and farmed fish [11,14]. Nifurtimox, another nitrofuran derivative, which was developed in the 1960s, has been used to treat Chagas disease caused by *T. cruzi*. More recently, the combination of nifurtimox and eflornithine was evaluated for the treatment of late-stage *T. b. gambiense* sleeping sickness [15,16,17]. Furthermore, a study revealed that chemically synthesized analogs of nitrofurantoin had significantly enhanced antimycobacterial activity [11]. Overall, these reports suggest that some nitrofurantoin analogs can be used against African trypanosomes. Therefore, the aim of the study was to evaluate the in vitro trypanocidal activity of these chemically synthesized nitrofurantoin analogs (Table 1) and in vivo treatment efficacy of three promising analogs against human and animal trypanosomes.

## 2. Results

### 2.1. In Vitro Experiment

A summary of the trypanocidal activity and cytotoxicity data of the nitrofurantoin analogs is shown in Table 2.

Trypanocidal activities differed among the trypanosome species. However, *T. congolense* IL3000 and *T. b. rhodesiense* Chirundu were more sensitive to these nitrofurantoin analogs than the other trypanosome strains/species.

The strongest trypanocidal activity (IC_50_ < 0.031 µM) against *T. congolense* IL3000 and *T. b. rhodesiense* Chirundu was observed with the long chain analogs **9**–**12**, bearing side chains of **9**–**11** carbons, respectively (*n* = 9–12), in sub-series 1. These analogs showed relatively moderate (0.16–0.65 µM) activity against *T. evansi* Tansui, *T. b. brucei* GUTat3.1, *T. b. gambiense* IL1922, and *T. b. rhodesiense* IL1501.

Furthermore, variation in the trypanocidal activity for analogs in sub-series 2 was found based on the electronegativity of *para*-substituents on the phenyl ring. The trypanocidal activity of analogs bearing electron-withdrawing groups (**16**, **17**, and **19)** was stronger than that of analog **14**, featuring a neutral group and those of analogs **15** and **18** possessing electron-donating groups. In contrast, electronegativity had no effect on cytotoxicity within sub-series 2.

### 2.2. In Vivo Experiment

**Figure 1:**
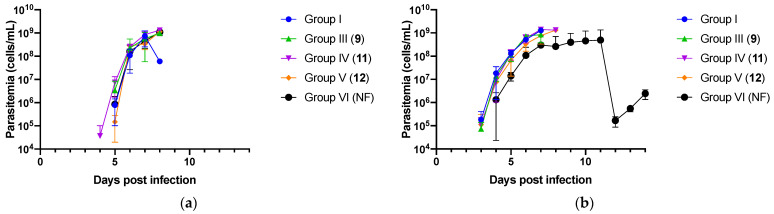
In vivo analysis of selected nitrofurantoin analogs and nitrofurantoin (NF). Evaluation of parasitemia in mice infected with *T. congolense* and intraperitoneal or orally treated with nitrofurantoin analogs (**9**, **11**, and **12**) and nitrofurantoin. The Y axis shows log 10 scale, and the data are shown as mean ± standard deviation. Parasitemia was not observed in group II (diminazene aceturate treatment). (**a**) Treatment of mice by intraperitoneal injection at 0.1 mg/kg. (**b**) Treatment of mice by oral administration at 10 mg/kg.

The in vivo trypanocidal activity of three selected nitrofurantoin analogs (**9**, **11**, and **12**) showed strong trypanocidal activity and were used to confirm the results of in vitro assay and nitrofurantoin. These three analogs were evaluated. All mice treated with nitrofurantoin and its analogs by intraperitoneal injection at 0.1 mg/kg, died within 9 dpi, because of high parasitemia (Figure 1a). All mice orally treated with the three analogs at 10 mg/kg also succumbed to high parasite levels within 9 dpi. In contrast, two of three mice orally treated at 10 mg/kg with nitrofurantoin, survived until end of the experiment period (Figure 1b).

## 3. Discussion

**Figure 2:**
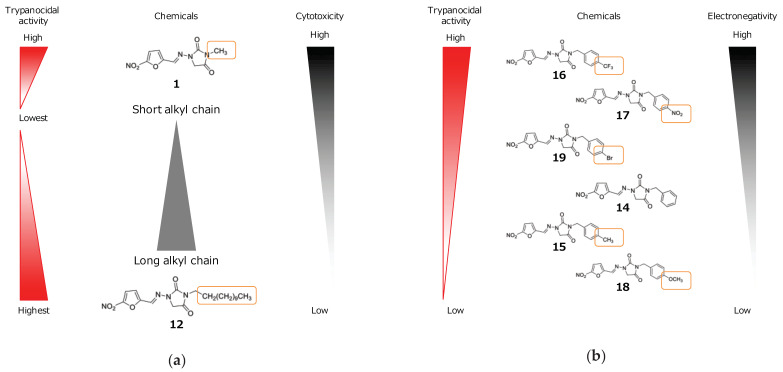
Summary of structure–activity relationship. The summary of structure–activity relationship between trypanocidal activity and alkyl chain length (**a**) or electronegativity (**b**).

In the present study, the trypanocidal activity of 19 nitrofurantoin analogs was evaluated against six human and animal trypanosomes in vitro. Investigation of the structure–activity relationship within the series revealed that the trypanocidal activity of short alkyl analogs (**1**–**4**) in sub-series 1 decreased with increasing chain lengths up to four carbons (*n* = 4, **4**) (Figure 2a). The trypanocidal activity in long alkyl chain analogs (**5**–**12**) increased with increasing chain lengths up to 12 carbons (*n* = 12, **12**) (Figure 2a). The cytotoxicity within the analogs of sub-series 1 mostly increased with increasing chain lengths (Figure 2a). The strongest trypanocidal activity of these long alkyl chains (**9**–**11**) in sub-series 1 may be related to their higher lipophilicity, which increased the cLogP value. This feature is typically associated with better permeation of a molecule through biological tissues/membranes, as well as interactions with transporter proteins and enzymes [18]. Similar results were previously observed against the *T. cruzi* parasite [19,20]. Various studies have indicated a relationship between trypanocidal activity and the number of carbon atoms in the alkyl chain of the compounds [21,22].

Additionally, we showed that the trypanocidal activity of analogs bearing electron-withdrawing groups (**16**, **17**, and **19**) was stronger than that of the compounds containing a neutral group (**14**) and those bearing electron-donating groups (**15** and **18**) in sub-series 2 (Figure 2b). Electronegativity was also reported to affect the trypanocidal activity [23,24,25]. Our results are similar to the previous findings, except for the differences in the values. The trypanocidal activity of the nitrofurantoin analogs increased with increasing chain length, up to a certain number of carbon atoms and electronegativity. Our results confirm previous findings and extend the range of promising compounds that are optimized by a combination of chemical modifications with long alkyl chain and high electronegativity. This can be strategized using a fragment-based, drug-discovery approach to enhance the trypanocidal activity of nitrofurantoin. The presence of an electronegative electron-withdrawing group in the *ortho*- or *meta*-position on the phenyl ring may also influence activity. However, these structure–activity relationships must be confirmed in further experiments by investigating derivatives bearing such substituents.

Furthermore, the selectivity index, which indicates the selectivity of the antipathogenic action of a compound in the presence of normal/healthy mammalian cells (Table 2), showed that most analogs had a selectivity index >10, which is a minimum criterion value for selecting a synthetic drug hit [26]. Therefore, most analogs were hits, with activity IC_50_ <10 µM and selectivity index >10 [26]. Despite being mild to weakly toxic towards Madin–Darby bovine kidney cells [27,28], these analogs were up to 10–100-fold selective in their trypanocidal action.

Hall et al. suggested that trypanosomal type I nitroreductase reduces nitrofuran compounds, and that the toxic intermediate metabolites kill the parasites [29]. They also revealed that an increased expression level of this enzyme makes *T. b. brucei* more sensitive to nifurtimox and its related compounds [12]. The current study revealed differences in the sensitivities of the evaluated trypanosome species and strains to the 19 nitrofurantoin analogs. This may explain the difference in the expression level of trypanosomal type I nitroreductase or enzymatic activity in each trypanosome, leading to differences in the sensitivity of the tested compounds.

Nitrofurantoin (Macrobid^®^) is an antibiotic that is orally used to treat bladder infections. Nitrofurantoin shows extremely low lipophilicity (clogP −0.22), which may explain its poor trypanocidal activity, as it cannot efficiently permeate parasitic biomembranes. In contrast, analogs **1** and **2** have low cLogP values of −0.04 and 0.36, respectively [11], which are relatively higher than those of nitrofurantoin, suggesting their moderately lipophilic nature. They were found to possess moderate (0.19–0.73 µM) activity against the *T. b. rhodesiense* Chirundu and *T. congolense* IL3000 strains. HAT and AT are endemic diseases, mainly occurring in rural areas of developing countries, and persist because of the lack of healthcare and veterinary medicine infrastructure. Therefore, oral drugs are highly convenient for treating these diseases. Although they showed strong trypanocidal activities in vitro, no treatment efficacy was observed with nitrofurantoin analogs **9**, **11**, and **12** against *T. congolense* infection in vivo, after both oral and intraperitoneal administration. Oral treatment with nitrofurantoin, however, demonstrated partial efficacy. nitrofurantoin possessed weak trypanocidal activity in vitro, but dissolved in water with a lower clogP than that of the tested analogs, which were more lipophilic and thus had higher clogP values. This result supports that high-hydrosolubility nitrofurantoin analogs should be developed and evaluated for their in vivo trypanocidal activity, in further studies. Analogs **1** and **2** showing moderate in vitro trypanocidal activity and higher solubility (lower cLogP) than the current selectees may be viable starting points for developing oral treatments for HAT and AT in endemic areas [30].

## 4. Materials and Methods

### 4.1. Chemicals

The nitrofurantoin analogs (Table 1) were synthesized in moderate yields (50–65%) in a single-step *N*-alkylation process through nucleophilic substitution (S_N_2) [11]. The series of analogs was divided into two sub-series based on the nature of the substituent at the N-3 position. Thus, sub-series 1 comprised N-alkyl analogs **1**–**12,** whereas sub-series 2 comprised benzyl-substituted analogs **13**–**19**. The latter sub-series featured analogs **15** and **18**, which bore the electron-donating groups CH_3_ and OCH_3_, respectively, whereas analogs **16**, **17,** and **19** possessed the electron-withdrawing groups CF_3_, NO_2_, and Br, respectively. Analog **14** bore a neutral group H, and analog **13** possessed an ethyl bridge between the nitrofurantoin scaffold and the benzyl group.

The analogs possessed lipophilic properties, with lipophilicity coefficients (clogP) in the targeted range of 1–5 [31,32]. They showed moderate to no toxicity in human embryonic kidney (HEK-293) and Chinese hamster ovary cells and exhibited anti-mycobacterial activity with 90% minimum inhibitory concentrations of 0.5–35 µM. The most potent anti-mycobacterial nitrofurantoin derivative was analog **8**, which was 30-fold more potent than nitrofurantoin [11].

These analogs were dissolved at 10 mg/mL in dimethyl sulfoxide and stored at −30 °C until use.

### 4.2. In Vitro Cultivation of Trypanosomes and Madin–Darby Bovine Kidney Cells

The bloodstream forms of *T. b. brucei* GUTat3.1, *T. b. rhodesiense* IL1501, *T. b. rhodesiense* Chirundu, *T. b. gambiense* IL1922, and *T. evansi* Tansui strains were propagated in culture at 37 °C, whereas the *T. congolense* IL3000 strain was propagated at 33 °C in Hirumi’s modified Iscove’s medium-9, supplemented with 20% heat-inactivated fetal bovine serum [33]. The bloodstream form cultures were maintained by replacing the entire culture supernatant with fresh medium, every other day.

### 4.3. Evaluation of Trypanocidal Activity and Cytotoxicity of Nitrofurantoin Analogs In Vitro

The trypanocidal activity and cytotoxicity of nitrofurantoin analogs were evaluated according to previous reports [34,35], with some modifications. In brief, bloodstream forms adjusted at 2.5 × 10^3^ cells/mL (*T. b. brucei* GUTat3.1, *T. b. gambiense* IL1922, and *T. b. rhodesiense* IL1501), 1 × 10^4^ cells/mL (*T. evansi* Tansui and *T. b. rhodesiense* Chirundu), and 1 × 10^5^ cells/mL (*T. congolense* IL3000), were cultivated in Nunc^®^ MicroWell 96-well optical bottom plates (Thermo Fisher Scientific, Waltham, MA, USA) and exposed to various analogs at seven different concentrations for 3 days, in an incubator at 37 °C (33 °C for *T. congolense* IL3000). After 3 days of incubation, the condition of the bloodstream forms was observed with phase-contrast microscopy. Twenty-five microliters of the Cell-TiterGlo^®^ (Promega, Madison, WI, USA) reagent was added into each well, and bioluminescence was measured using a GloMax^®^-Multi+Detection System plate reader (Promega).

Additionally, Madin–Darby bovine kidney cells were cultivated (density, 1 × 10^4^ cells/mL) in a 96-well cell culture plate (Thermo Fisher Scientific) with the analogs, at seven different concentrations for 3 days at 37 °C. Next, 10 µl of the Cell Counting Kit-8 solution (Dojindo, Kumamoto, Japan) was added into each well before and after 4 h of incubation. Absorbance was measured at 450 nm using a GloMax^®^-Multi+Detection System plate reader.

The half maximum inhibitory concentration (IC_50_) of each analog in trypanosomes and the Madin–Darby bovine kidney cells was calculated using the GraphPad Prism version 8 software (GraphPad, Inc., San Diego, CA, USA).

### 4.4. In Vivo Evaluation of the Treatment Efficacy of Selected Nitrofurantoin Analogs

Healthy female 8-week-old BALB/c mice (CLEA Japan, Inc., Tokyo, Japan) were used in this study. All animals had ad libitum access to normal chow and water.

The virulent *T. congolense* IL3000 strain was propagated in a mouse and used for infection. The parasites were passaged once in a mouse before the experiment. The experimental mice were intraperitoneally infected with 100 μL of *T. congolense* in phosphate-buffered saline, containing 10% glucose at 1 × 10^3^ cells/mouse. First, the mice were randomly divided into six groups of three mice, each as follows. Group I (no treatment), mice were not infected and were only treated with solvent (10% dimethyl sulfoxide–phosphate–buffered saline). Group II received 3.5 mg/kg of diminazene aceturate treatment. Groups III (**9**), IV (**11**), V (**12**), and VI were treated with nitrofurantoin and the mice were infected and intraperitoneally treated with 0.1 mg/kg of the compound.

In the second experiment, the mice were randomly divided into six groups of three mice, each as follows—group I received no treatment; group II received 3.5 mg/kg of diminazene aceturate treatment; and groups III (**9**), IV (**11**), V (**12**), and VI received nitrofurantoin—the mice were infected and orally treated with 10 mg/kg of the compound.

Treatment was initiated at 2 days post-infection (dpi) and continued for seven consecutive days. The treatments were freshly prepared each day. Parasitemia was observed using a cell counting chamber until 14 dpi.

## 5. Conclusions

In conclusion, our results indicate that nitrofurantoin may be suitable as a lead compound for developing novel trypanocidal drugs, provided that compounds with good oral bioavailability and in vivo activity can be designed, particularly against animal African trypanosomosis caused by *T. congolense* and some confined endemic acute HAT infections caused by the *T. b. rhodesiense* Chirundu strain. Our preliminary in vivo experiments also showed that NF and its analogs with high water solubility should be evaluated to determine their treatment efficacy in mouse models, in further studies.

## Figures and Tables

**Table 1:** Structures of synthesized nitrofurantoin analogues **1**–**19**.

Cpd.	MW (g/mol)	Name	Structure
**1**	252.18	(*E*)-3-Methyl-1-([(5-nitrofuran-2-yl) methylene]amino)imidazolidine-2,4-dione	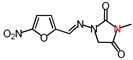
**2**	266.21	(*E*)-3-Ethyl-1-([(5-nitrofuran-2-yl) methylene]amino)imidazolidine-2,4-dione	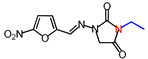
**3**	280.24	(*E*)-1-([(5-Nitrofuran-2-yl) methylene]amino)-3-propylimidazolidine-2,4-dione	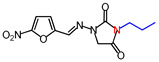
**4**	294.26	(*E*)-3-Butyl-1-([(5-nitrofuran-2-yl) methylene]amino)imidazolidine-2,4-dione	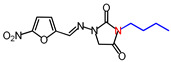
**5**	30,829	(*E*)-1-([(5-Nitrofuran-2-yl)methylene] amino)-3-pentylimidazolidine-2,4-dione	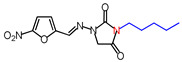
**6**	322.32	(*E*)-3-Hexyl-1-([(5-nitrofuran-2-yl) methylene]amino)imidazolidine-2,4-dione	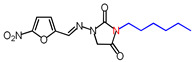
**7**	336.34	(*E*)-3-Heptyl-1-([(5-nitrofuran-2-yl) methylene]amino)imidazolidine-2,4-dione	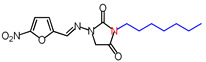
**8**	350.37	(*E*)-1-([(5-Nitrofuran-2-yl)methylene] amino)-3-octylimidazolidine-2,4-dione	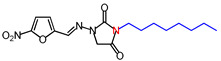
**9**	364.4	(*E*)-1-([(5-Nitrofuran-2-yl)methylene] amino)-3-nonylimidazolidine-2,4-dione	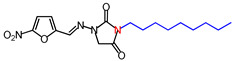
**10**	378.42	(*E*)-3-Decyl-1-([(5-nitrofuran-2-yl)methylene] amino)imidazolidine-2,4-dione	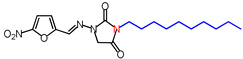
**11**	392.45	(*E*)-1-([(5-Nitrofuran-2-yl)methylene] amino)-3-undecylimidazolidine-2,4-dione	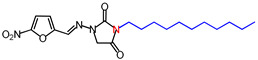
**12**	406.48	(*E*)-3-Dodecyl-1-([(5-nitrofuran-2-yl) methylene]amino)imidazolidine-2,4-dione	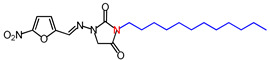
**13**	356.33	(*E*)-1-([(5-Nitrofuran-2-yl)methylene] amino)-3-(3-phenylpropyl)imidazolidine-2,4-dione	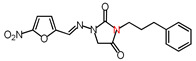
**14**	328.28	(*E*)-3-Benzyl-1-([(5-nitrofuran-2-yl) methylene]amino)imidazolidine-2,4-dione	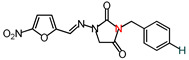
**15**	342.31	(*E*)-3-(*p*-Methylbenzyl)-1-([(5-nitrofuran-2-yl) methylene]amino)imidazolidine-2,4-dione	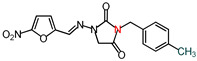
**16**	396.28	(*E*)-1-([(5-Nitrofuran-2-yl)methylene] amino)-3-(*p*-(trifluoromethyl)benzyl) imidazolidine-2,4-dione	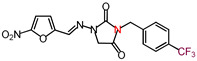
**17**	373.28	(*E*)-3-(*p*-Nitrobenzyl)-1-([(5-nitrofuran-2-yl) methylene]amino)imidazolidine-2,4-dione	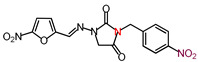
**18**	358.31	(*E*)-3-(*p*-Methoxybenzyl)-1-([(5-nitrofuran-2-yl) methylene]amino)imidazolidine-2,4-dione	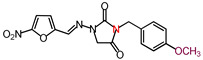
**19**	407.18	(*E*)-3-(*p*-Bromobenzyl)-1-([(5-nitrofuran-2-yl) methylene]amino)imidazolidine-2,4-dione	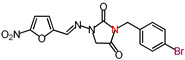

**Table 2:** In vitro biological activities of synthesized nitrofurantoin analogs.

			Trypanocidal Activity IC_50_ ± SD (µM) ^c^						Cytotoxicity IC_50_ ± SD (µM) ^c^	Selectivity Index (IC_50_ of Trypanosomes/IC_50_ of MDBK) ^d^					
ID	N ^a^	clogP ^b^	Tbb GUTat3.1	Tbg IL1922	Tbr IL1501	Tbr Chirundu	Tc IL3000	Tev Tansui	MDBK	Tbb GUTat3.1	Tbg IL1922	Tbr IL1501	Tbr Chirundu	Tc IL3000	Tev Tansui
**1**	1	−0.04	3.61 ± 0.40	3.60 ± 0.26	7.02 ± 0.094	0.73 ± 0.17	0.19 ± 0.076	1.61 ± 0.098	65.21 ± 12.78	18	18	9	89	342	41
**2**	2	0.36	10.81 ± 4.84	11.31 ± 4.80	11.40 ± 5.24	0.51 ± 0.25	0.42 ± 0.16	5.39 ± 0.53	44.79 ± 1.69	4	4	4	87	108	8
**3**	3	0.88	14.13 ± 0.34	14.73 ± 1.32	14.02 ± 1.42	0.81 ± 0.24	0.33 ± 0.18	6.53 ± 0.61	39.29 ± 1.09	3	3	3	49	120	6
**4**	4	1.33	33.29 ± 24.29	17.25 ± 2.23	32.74 ± 0.82	1.38 ± 0.34	0.31 ± 0.20	7.89 ± 0.53	35.78 ± 3.49	1	2	1	26	117	5
**5**	5	1.77	4.24 ± 1.99	3.42 ± 1.47	5.69 ± 1.66	NA	0.35 ± 0.15	5.44 ± 0.17	39.07 ± 5.86	9	11	7	NA	112	7
**6**	6	2.22	5.76 ± 4.01	2.59 ± 0.28	2.61 ± 0.12	0.17 ± 0.11	0.08 ± 0.047	3.76 ± 0.47	25.18 ± 3.52	4	10	10	146	321	7
**7**	7	2.66	2.06 ± 0.75	1.99 ± 0.75	1.45 ± 0.86	0.056 ± 0.026	0.040 ± 0.011	1.02 ± 0.10	92.28 ± 6.52	45	46	64	1654	2323	90
**8**	8	3.10	1.89 ± 0.69	1.40 ± 0.73	1.38 ± 0.82	0.039 ± 0.0084	0.030 ± 0.0050	0.81 ± 0.082	84.74 ± 8.99	45	61	62	2195	2809	105
**9**	9	3.55	0.57 ± 0.22	0.44 ± 0.17	0.60 ± 0.54	0.017 ± 0.0067	0.010 ± 0.0035	0.27 ± 0.025	251.18 ± 58.27	443	567	420	14,645	25,639	918
**10**	10	3.99	0.65 ± 0.56	0.62 ± 0.70	0.61 ± 0.06	0.017 ± 0.0044	0.011 ± 0.0044	0.28 ± 0.025	110.45 ± 14.38	170	177	180	6645	10,502	402
**11**	11	4.44	0.33 ± 0.12	0.33 ± 0.12	0.24 ± 0.13	0.021 ± 0.0094	0.011 ± 0.0035	0.16 ± 0.014	>254.81	764	771	1052	11,933	21,978	1602
**12**	12	4.88	0.34 ± 0.12	0.23 ± 0.13	0.26 ± 0.11	0.031 ± 0.011	0.012 ± 0.0045	0.20 ± 0.017	>246.02	715	1095	934	7937	21,322	1253
**13**		2.46	7.29 ± 3.16	4.83 ± 3.22	5.90 ± 3.75	0.14 ± 0.067	0.14 ± 0.030	3.11 ± 0.37	>280.64	39	58	48	2,041	2058	90
**14**		1.73	2.88 ± 0.26	2.34 ± 0.64	2.83 ± 0.61	0.27 ± 0.12	0.18 ± 0.017	1.26 ± 0.10	>304.62	106	130	108	1142	1702	241
**15**		2.24	8.24 ± 3.11	2.89 ± 0.51	6.10 ± 0.047	0.15 ± 0.068	0.13 ± 0.044	4.23 ± 0.40	34.28 ± 11.5	4	12	6	231	271	8
**16**		2.60	0.52 ± 0.081	0.61 ± 0.26	0.96 ± 0.0087	0.02 ± 0.012	0.02 ± 0.073	0.44 ± 0.046	56.62 ± 40.76	108	93	59	3121	3802	128
**17**		1.67	1.07 ± 0.74	1.05 ± 0.70	0.50 ± 0.012	0.04 ± 0.094	0.04 ± 0.017	0.61 ± 0.050	20.10 ± 1.02	19	19	45	497	469	33
**18**		1.57	9.91 ± 0.092	9.78 ± 0.31	10.14 ± 0.62	0.33 ± 0.22	0.14 ± 0.030	2.40 ± 0.19	80.09 ± 36.89	8	8	8	244	585	33
**19**		2.50	2.13 ± 0.23	2.04 ± 0.091	2.47 ± 0.69	0.041 ± 0.025	0.049 ± 0.033	1.15 ± 0.11	28.74 ± 5.69	14	14	12	700	587	25
Nitrofurantoin		−0.22	1.42 ± 0.15	1.62 ± 0.28	1.42 ± 0.25	NA	0.36 ± 0.043	1.96 ± 0.50							
Nifurtimox			4.66 ± 19.7	4.58 ± 2.38	4.35 ± 1.59	1.46 ± 0.35	1.06 ± 0.22	2.62 ± 1.40							
Eflornithine			38.56 ± 9.88	36.66 ± 12.87	45.99 ± 17.07	35.65 ± 10.16	16.13 ± 2.93	57.21 ± 17.56							
Pentamidine			0.041 ± 0.0023	0.014 ± 0.0031	0.029 ± 0.0062	0.050 ± 0.012	0.33 ± 0.054	0.00097 ± 0.00019							
Suramin			0.066 ± 0.0052	0.064 ± 0.0018	0.076 ± 0.011	0.066 ± 0.071	7.17 ± 0.87	0.38 ± 0.058							

^a^ number of carbon atoms in alkyl side chain. ^b^ clogP of compounds D of compounds were referred to Zuma et al. [11]. ^c^ represents the mean ± the standard deviation (SD) from triplicate biological experiments. ^d^ value as rounded off to the nearest digit. NA—not analyzed.

## Data Availability

Not applicable.

## Sample Availability

Samples of the compounds are available from D.D.N.
